# Sex-based immunological differences in multisystem inflammatory syndrome in children: potential role of T_R3–56_ cells for pathogenesis, diagnosis, and therapy

**DOI:** 10.3389/fimmu.2025.1606115

**Published:** 2025-06-20

**Authors:** Flavia Carriero, Monica Gelzo, Valentina Rubino, Giulia Scalia, Alice Castaldo, Vincenzo Tipo, Antonietta Giannattasio, Carolina D’Anna, Giuseppina Ruggiero, Giuseppe Castaldo, Giuseppe Terrazzano

**Affiliations:** ^1^ Department of Health Sciences, University of Basilicata, Potenza, Italy; ^2^ Centro di Ingegneria Genetica e Biotecnologie Avanzate F. Salvatore (CEINGE) Advanced Biotechnology Franco Salvatore, Naples, Italy; ^3^ Department of Molecular Medicine and Medical Biotechnology, University of Naples Federico II, Naples, Italy; ^4^ Department of Translational Medical Sciences, University of Naples Federico II, Naples, Italy; ^5^ Azienda Ospedaliera di Rilievo Nazionale (AORN) Santobono-Pausilipon Hospital, Naples, Italy

**Keywords:** MIS-C, immune regulation, Treg, T_R3-56_ cells, biomarkers, sex-based differences

## Abstract

Multisystem Inflammatory Syndrome in Children (MIS-C) is characterized by immune dysregulation, exhibiting clinical and immunological features reminiscent of autoimmune processes, although its underlying mechanisms remain incompletely understood. This study examines immune system alterations in MIS-C patients, focusing on T_R3–56_ lymphocytes, a novel population of regulatory T cells. Our findings reveal a positive correlation between circulating T_R3–56_ cells and regulatory T cells, suggesting a potential immunoregulatory role in MIS-C pathogenesis. Furthermore, we identified significant sex-based differences in immune responses. Male patients exhibit higher percentages of T_R3–56_ lymphocytes and increased expression of T cell activation markers, which correlate with greater disease severity. Conversely, female patients display immune profiles characterized by stronger immune T cell memory and regulatory responses, potentially helping to modulate inflammation. These findings highlight the relevance of considering sex-based differences in immune responses to MIS-C and suggest that T_R3–56_ lymphocytes may serve as novel biomarkers and potentially as therapeutic targets. Our study enhances the understanding of immune dysregulation in MIS-C and underscores the need for sex-specific therapeutic strategies to improve patient outcomes.

## 1 Introduction

Multisystem Inflammatory Syndrome in Children (MIS-C) is a rare, severe condition that predominantly affects children and adolescents (<18 years), typically occurring 2 to 6 weeks after the acute infection of Severe Acute Respiratory Syndrome COronaVirus 2 (SARS-CoV-2) (1, 2).

Notably, the age group most frequently affected by this syndrome is children aged 5–13 years, although some data show an even wider range (from 1.6 up to 20 years) (1–5).

MIS-C clinically presents with a multi-system involvement, featuring persistent fever, gastrointestinal, mucocutaneous, cardiovascular (e.g., hypotension, shock), and neurological symptoms, along with potential mild respiratory involvement and a diffuse maculopapular rash (1–5). Diagnosis is essentially based on clinical criteria, elevated inflammatory markers, multisystem involvement, and evidence of recent SARS-CoV-2 exposure (6).

Treatment aims to control inflammation, support organ function, and manage complications with immunomodulatory therapies (e.g., intravenous immunoglobulin, corticosteroids, biologics), anticoagulation for thrombotic risks, and supportive care (7–9). The prognosis for children with MIS-C is generally favorable, with the majority achieving complete recovery following timely therapeutic intervention. Nevertheless, a subset of patients experiencing severe disease may develop long-term cardiac complications (7–9). The overall mortality associated with MIS-C remains below 2% (1–5, 7–9).

The pathophysiology of MIS-C is incompletely understood, but it is believed to result from an exaggerated immune response to the SARS-CoV-2, characterized by a hyperinflammatory state and dysregulated immune response marked by elevated cytokines [such as Interleukin (IL)-6, IL-1β, Interferon (INF)-γ and Tumor Necrosis Factor (TNF)-α], inflammatory markers (such as CRP and ferritin) alongside an overactivation of the innate and adaptive immune systems (10–14).

Studies have revealed significant activation of T cells, as well as the presence of autoantibodies, suggesting an autoimmune-like component (10–13). The T cells that remained often exhibited signs of heightened activation, evidenced by increased expression of Human Leukocyte Antigen (HLA)-DR (15–17).

In light of the evidence of immune hyperactivation in MIS-C, it is of particular interest to investigate potential alterations in immune regulation associated with the syndrome.

Immune regulation achieves a balanced response through a complex network of cells and molecules that distinguish self from non-self (18).

Crucial to immune regulation, FoxP3^+^ regulatory T cells (Tregs) maintain immune homeostasis by controlling responses, preventing autoimmunity, and limiting overreactions to infections (19–21). Dysfunctional Tregs disrupt this balance, potentially causing autoimmune diseases through excessive immune activity or weakened immunity through over-suppression (19–27).

Within the framework of immune regulation, we previously identified a novel potential regulatory cell population, CD3^+^ CD56^+^ T cells (T_R3-56_) (28).

In type 1 diabetes (T1D), lower T_R3–56_ cell frequencies correlate with increased cytotoxic CD8^+^ T lymphocyte (CTL) activation, worse disease progression, impaired β-cell function, and diabetic ketoacidosis (28). T_R3–56_ suppress CTL activity via reactive oxygen species reduction, but this mechanism is impaired in T1D, highlighting their role as key CTL regulators and potential T1D self-tolerance biomarkers (28). Similarly, T_R3–56_ cells negatively correlate with CTLs in myelodysplastic syndromes (MDS) (29, 30) and may contribute to immune escape in chronic lymphocytic leukemia (CLL) (31). In COVID-19, T_R3–56_ cells exhibit both regulatory and effector functions, modulating inflammation, potentially limiting tissue damage, and promoting immune balance (32).

An intriguing aspect of immune responses and immune regulation is the difference between male and female individuals (33). In this regard, sex-based variations in immune function are influenced by both sex hormones and the presence of two X chromosomes in females (33–36). Compared to males, females typically exhibit stronger innate and adaptive immune responses (33–36). Conversely, males tend to have higher infection-related mortality (33–36).

It is of some relevance that MIS-C exhibits gender-based differences, with male children showing a higher incidence and more severe outcomes than females (1–5, 7–9, 37). Males are at higher risk of developing MIS-C after SARS-CoV-2 infection and typically exhibit more severe symptoms, whereas females are less frequently affected and tend to present with milder clinical manifestations (37–39). In the context of previous coronavirus epidemics, such as Severe Acute Respiratory Syndrome-Coronavirus (SARS-CoV) and Middle East Respiratory Syndrome-Coronavirus (MERS-CoV), studies have shown that sex differences affect disease severity and clinical outcomes (37–39). The underlying mechanisms driving these gender differences are not fully understood but are thought to involve a combination of hormonal, immune, and genetic factors that differ between males and females (33–39).

In this scenario, we previously identified that MIS-C patients exhibit elevated levels of key cytokines, including IFN-γ, IL-6, IL-10, and TNF-α (12). Furthermore, dysregulation was observed in lymphocyte subpopulations, including NK cells, B cells, T cells, Th1, and Th17 cells, but no significant differences in Treg populations between MIS-C patients and healthy controls.

The objective of this current investigation was to elucidate the immunological mechanisms contributing to MIS-C, with a specific focus on immune cell dysregulation and the involvement of T_R3–56_ cells in its pathogenesis. A detailed analysis of the immunological characteristics of our previously characterized MIS-C patient cohort (12) was conducted. The primary aims were to assess the frequency of T_R3–56_ lymphocytes and their potential role in immune regulation in the context of MIS-C. Additionally, sex-based differences in immune profiles between male and female MIS-C patients were explored.

## 2 Materials and methods

### 2.1 Study population

Between March 2021 and March 2022, we prospectively enrolled pediatric patients who fulfilled the diagnostic criteria for MIS-C at the time of hospital admission, as defined by the CDC, the World WHO, and other relevant references (40, 41). Disease severity was assessed based on the extent of multisystem involvement, the specific organs affected, and the requirement for advanced supportive therapies. Patients were classified as having severe MIS-C if they presented with critical manifestations such as cardiovascular shock, myocarditis, or the need for vasopressor and/or inotropic support. In contrast, moderate cases were defined by significant, yet non-life-threatening, multisystem involvement without evidence of hemodynamic instability or organ failure.

In accordance with established diagnostic criteria, evidence of recent SARS-CoV-2 infection or known exposure within four weeks prior to symptom onset was required, provided no alternative plausible diagnosis could explain the clinical presentation. Although RT-PCR testing of nasopharyngeal swabs was negative in all cases at the time of admission, variant-specific virological data were not available. Nonetheless, during the enrollment period, the B.1.617.2 (Delta) variant was the predominant SARS-CoV-2 strain circulating in Italy.

The study was approved by the Ethics Committee of the University of Naples Federico II, and all procedures adhered to the principles outlined in the Declaration of Helsinki. Written informed consent was obtained from each participant’s parent or legal guardian. The only exclusion criterion was the inability to obtain informed consent, which did not apply to any participant (n = 0).

The current study included 39 MIS-C patients (14 females and 25 males) with a mean age of 8 years (range 1–14 years) and an average T_R3–56_ percentage of 2.4% (range 0.1–21).

The control group consisted of 13 pediatric healthy individuals (9 females and 4 males) with a mean age of 7 years (range 0–13 years) and an average T_R3–56_ percentage of 2.0% (range 0.9–5.6).

Patients were studied at time of enrolment, prior to any therapeutic intervention in MIS-C patients that could modify the immune response.

All the patients recruited in the study cohort and belonging to the control group were of European origin (12) and recruited at the same time period (between March 2021 and March 2022).

### 2.2 Blood samples, immune phenotype and flow cytometry

Blood samples were obtained upon admission and collected in EDTA-containing tubes. Leukocyte counts were initially assessed using a hemocytometer and subsequently analyzed through multi-color flow cytometry with a FACS Canto II (Becton Dickinson).

Peripheral blood mononuclear cells (PBMC) were isolated by centrifugation of the peripheral blood on a Ficoll-Paque cushion (GE Healthcare, Uppsala, Sweden) gradient, as reported (42).

All phenotypes referred to flow cytometry analysis of the lymphocyte population gated by using Forward Scatter (FSC) and Side Scatter (SSC) parameters in PBMC (42).

Details regarding the lymphocyte subsets, their surface markers, the monoclonal antibodies (MoAB) and the fluorochromes used in the analysis have been previously documented (12) and reported in supplementary **Table 1**. T_R3–56_ lymphocytes have been identified by the co-staining with anti-human CD3 and anti-human CD56 mAb, as described (28). Gating strategy is reported in **Supplementary Figure 1**.

**Table 1:** Correlation between T_R3–56_ or Treg cells and immune cells in male and female MIS-C patients.

% T_R3–56_ cells		
Correlations in male	Slope	p Value
% CD45 RA^+^ naïve T lymphocytes	-0,5385	0,0143
% Activated Th1 lymphocytes	0,5019	0,0286
Correlations in female	Slope	p Value
% CD45 RA^+^ naïve T lymphocytes	-0,7178	0,0107
% CD45 RO^+^ memory T lymphocytes	0,7289	0,0091

% Treg cells		
Correlations in male	Slope	p Value
% Activated Th1 lymphocytes	0,4956	0,0309
Correlations in female	Slope	p Value
% Th1 lymphocytes	-0,7801	0,0040
CD4/CD8 T cells ratio	0,6415	0,0276

Spearman’s rank correlation is reported as Slope and p value, as indicated.

For serum cytokine, quantification, blood samples were drawn into tubes without anticoagulant, and the concentrations of IFN-α, IFN-β, IFN-γ, interleukin (IL)-6, IL-10, IL-17A, IL-12p70, and tumor necrosis factor (TNF)-α were measured using automated microfluidic immunoassay cartridges on the *ProteinSimple* Ella system (Bio-Techne), following the manufacturer’s guidelines.

### 2.3 Statistical analysis

Statistical analyses were performed using the *Mann-Whitney test* to compare differences between two independent groups, given the non-parametric nature of the data. Correlations between variables were assessed using *Spearman’s rank correlation* coefficient, which evaluates monotonic relationships without assuming a normal distribution. All statistical tests were two-tailed, and significance was set at p < 0.05. Analyses were conducted using Prism 9 GraphPad Inc. (San Diego, CA, USA).

## 3 Results

### 3.1 Comparison of T_R3–56_ cell level between MISC patients and control group of healthy subjects

**Figure 1:**
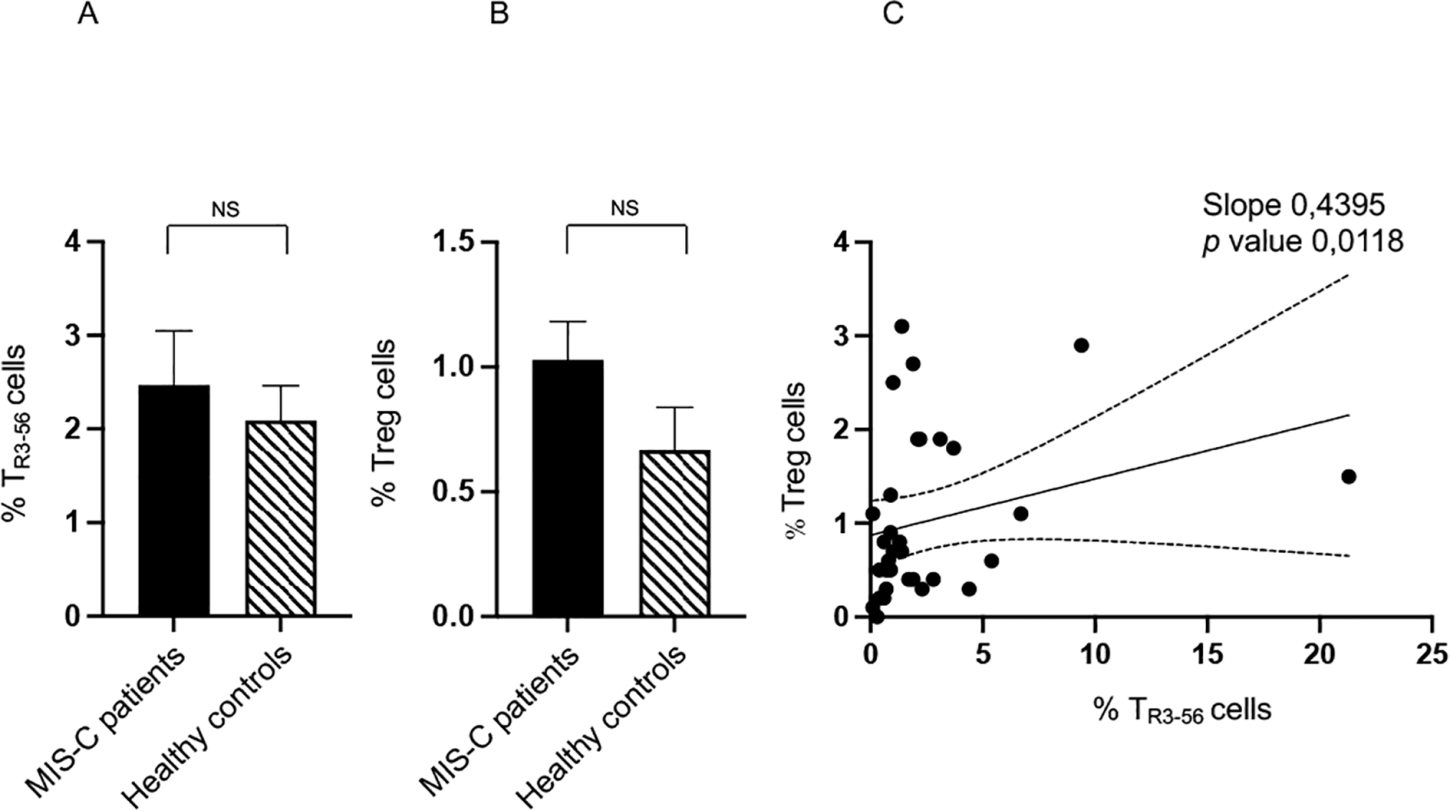
Comparison of T_R3–56_ cell level between MISC patients and control group of healthy subjects. Analysis of T_R3–56_ lymphocytes **(A)** and of Tregs **(B)** in MIS-C patient (black bars) and healthy controls (dashed bars). Cell percentages are reported in y axis. **(C)** Correlation between T_R3–56_ and Tregs in MIS-C patients. NS means not statistically significant. Spearman’s rank correlation is reported as Slope and *p* value.

We compared T_R3–56_ cell levels between MIS-C patients and healthy controls. Statistical evaluation revealed no significant differences in the percentages of T_R3–56_ lymphocytes (**Figure 1A**). Similarly, in line with our previous study (12), no differences were observed in the percentage of Tregs between MIS-C patients and healthy controls (**Figure 1B**). No significant differences were observed in the absolute counts of key effector cell populations - including T cells, B cells, and NK cells - between MIS-C patients and healthy controls (data not shown).

These results indicate that major quantitative alterations in T_R3-56_, Treg and other immune effector cell populations may not be a distinctive feature of MIS-C subjects. However, a trend towards higher percentages of both cell types was observed in the MIS-C group compared to the healthy individuals. Although this trend did not reach statistical significance, it could suggest a potential attempt to negatively modulate immune response in MIS-C.

However, significant alterations were identified in the correlation patterns among these immunocompetent cell populations. These disrupted associations point to potential dysregulation of immune network dynamics in MIS-C, occurring independently of changes in absolute cell counts.

In this regard, we observed a positive correlation between T_R3–56_ and Tregs in MIS-C patients (**Figure 1C**), absent in controls (data not shown). This evidence suggests a potential immunoregulatory role for T_R3–56_ in this disease context and may reflect either a compensatory regulatory mechanism or a feature of immune dysregulation characteristic of MIS-C.

### 3.2 Correlation between T_R3–56_ and immune profile in MISC patients and control group

We aimed to evaluate if the T_R3–56_ cells within MIS-C patients correlate with immunological markers of immune dysregulation, by conducting a correlation study between T_R3–56_ cells and relevant immune cell populations.

In this regard, T_R3–56_ lymphocyte percentages exhibited significant negative correlations with both CD19^+^ B cells (43) and CD45RA^+^ naïve T cells (44) (**Table 1**). CD45RA^+^ naïve T cells are described to be essential for immune responses against novel pathogens and to exhibit high proliferative potential upon antigen stimulation, undergoing activation and differentiation into effector and memory T cells (44).

The observed inverse relationships between T_R3–56_ and the above-described cell populations may indicate a potential role for T_R3–56_ cells in modulating early-stage immune activation and naïve lymphocyte homeostasis in MIS-C.

Conversely, T_R3–56_ levels showed positive correlations with CD45RO^+^ memory T cells (44), suggesting an association with antigen-primed T cell populations (**Table 1**).

Indeed, memory T cells arise following antigen exposure and differentiation from naïve T cells and contribute to long-term immunity (44).

Furthermore, T_R3–56_ percentages were positively correlated with activated Th1 cells (45), implying a potential involvement in immune regulation of pro-inflammatory responses (**Table 1**).

Finally, it is interesting to note that Tregs correlate with Th17 cells (**Table 1**). The latter cells play a dual role in immunity: they are essential for host defines, but their dysregulation contributes to autoimmune and inflammatory pathologies (45).

Notably, all these correlation patterns were absent in the control group (data not shown), underscoring a potential disease-specific immune signature in MIS-C patients.

### 3.3 Sex stratification in immune profile of MIS-C patients and correlations between T_R3–56_ cells and other immune cells

Consistent with previous studies reporting a higher prevalence of males in MIS-C cases (1–4, 7–9, 37), our cohort also exhibits a male predominance, with 25 males and 14 females.

Regarding disease severity, 14 of the 25 males present the disease with severe (6 individuals) or moderate (8 individuals) clinical manifestations, while 8 of the 14 females exhibit severe (3 individuals) or moderate (5 individuals) conditions.

To identify potential sex-based differences in MIS-C, we compared patterns of immune activation and regulation between male and female patients within our MIS-C cohort.

While our previous study (12) showed higher percentages of Natural Killer (NK) (46), B cells, T, and Th17 lymphocytes (45) in MIS-C patients relative to healthy controls, our current analysis revealed no significant sex-based differences in these cell populations within our MIS-C cohort (**Supplementary Figures 2A–F**).

**Figure 2:**
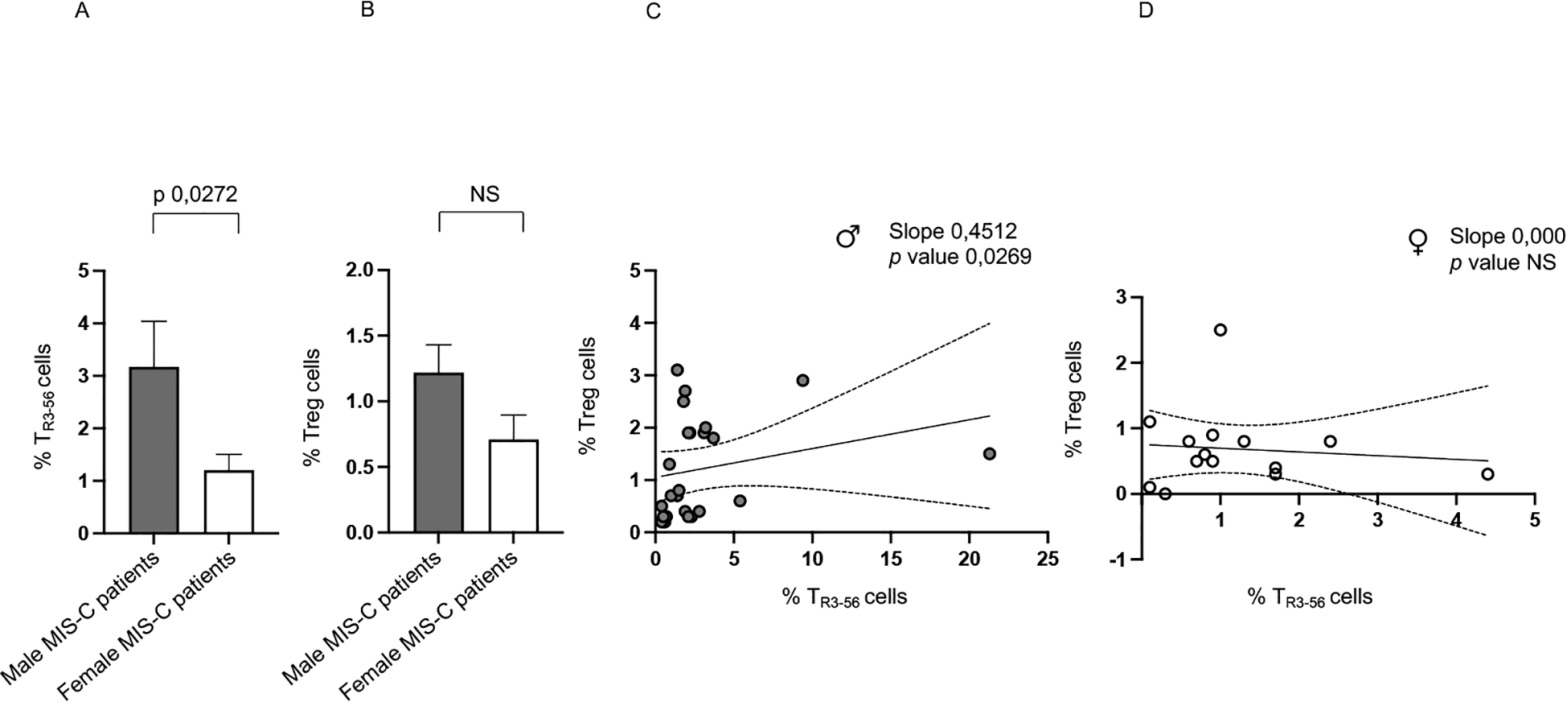
Sex-based differences in MIS-C patients. Analysis of T_R3-56_ **(A)** and Treg **(B)** cells in male (gray bars) and female (white bars) MIS-C patients. Cell percentages are reported in y axis. Correlations between T_R3–56_ and Treg cells in male ♂ **(C)** and female ♀ **(D)** MIS-C patients. Spearman’s rank correlation is reported as Slope and *p* value.

Notably, male MIS-C patients exhibit significantly higher percentages of T_R3–56_ cells compared to females (**Figure 2A**). While no significant sex-related differences are observed in Treg levels, there is a tendency toward higher Treg presence in males (**Figure 2B**).

In addition, it is relevant that a positive correlation between Tregs and T_R3–56_ is observed in males but not in females (**Figure 2C, D**, respectively).

These findings highlight a potential sex-based difference in the immune response of MIS-C, suggesting a more prominent role for T_R3–56_ cells in males and a potential sex-specific regulatory mechanism.

**Figure 3:**
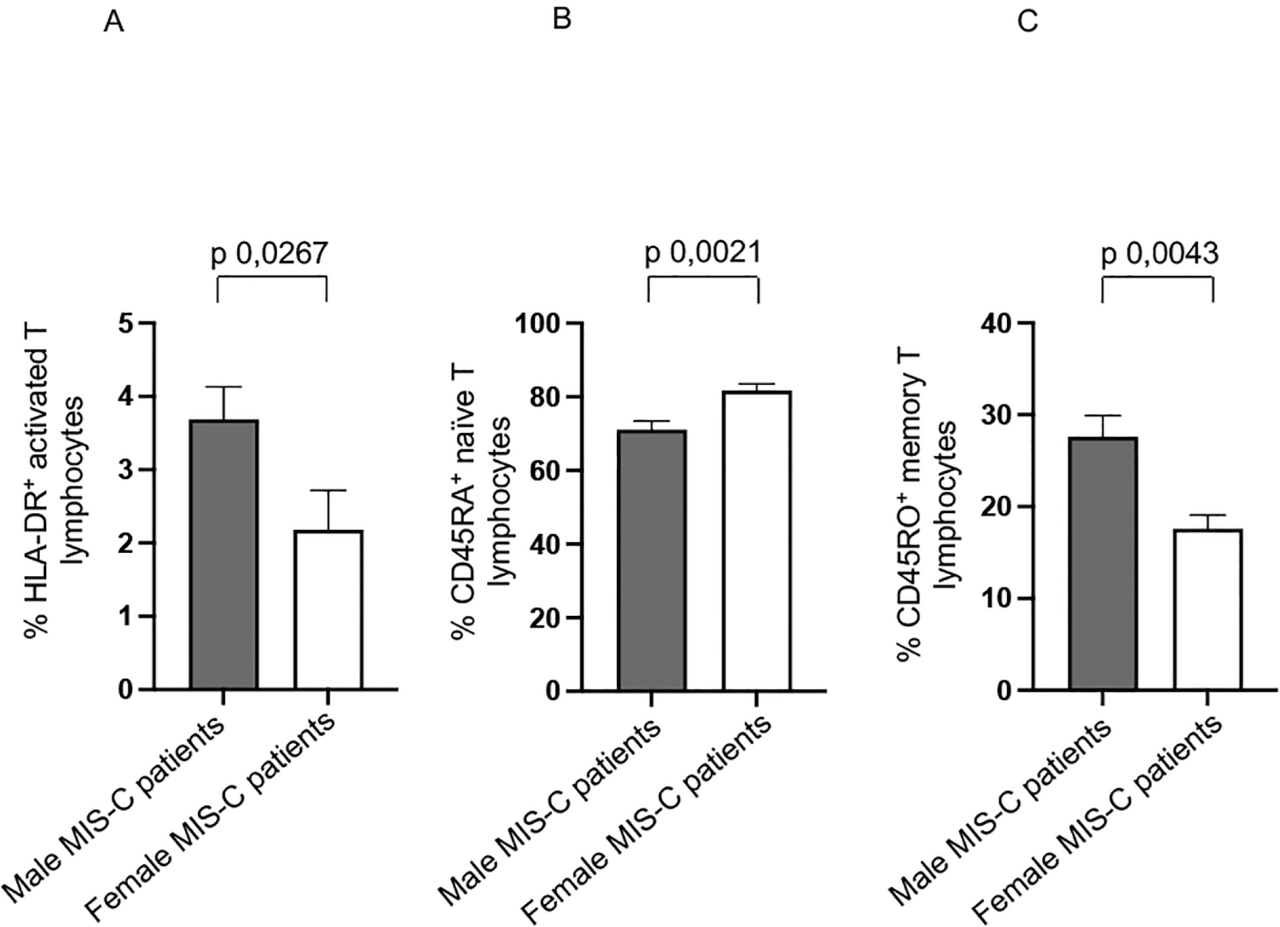
Other sex-based differences in MIS-C patients. Analysis of HLA-DR+ activated T **(A)**, CD45RA^+^ naïve T **(B)**, and CD45RO^+^ memory T **(C)** lymphocytes in male (gray bars) and female (white bars) MIS-C patients. Cell percentages are reported in y axis*. p* value is reported at the top of the bars. NS means not statistically significant.

However, it is noteworthy that male MIS-C patients exhibited significantly higher percentages of HLA-DR^+^ activated T (15–17, 47), and CD45RO^+^ memory T cells (44) (**Figures 3A, C**, respectively). We observed a higher percentage of CD45RA^+^ naïve T cells in female MIS-C patients (**Figure 3B**).

These results suggest a potentially heightened immune response in male MIS-C patients. Indeed, the increased presence of activated T cells and memory T cells could be indicative of a more pronounced inflammatory or adaptive immune response.

**Figure 4:**
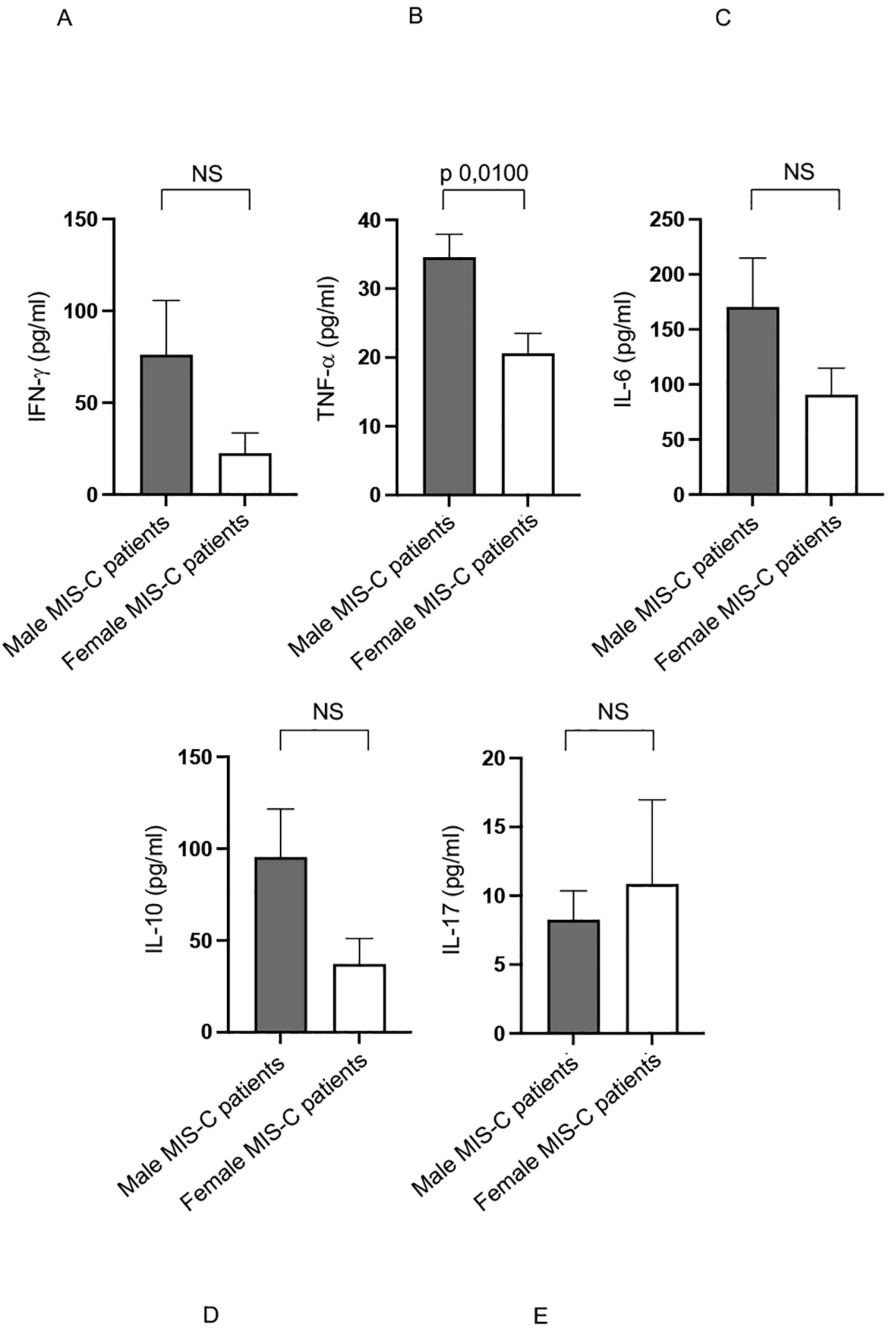
Cytokine productions in male and female MIS-C patients. Analysis of INF-γ **(A)**, TNF-α **(B)**, IL-6 **(C)**, IL-10 **(D)** and IL-17 **(E)** production in male (gray bars) and female (white bars) MIS-C patients. Cytokine amount values are reported in y axis. *p* value is reported at the top of the bars. NS means not statistically significant.

We previously observed a higher production of several cytokines in MIS-C patients if compared with healthy subjects (12). Analysis of the same cytokine production in sex-stratified MIS-C patients reveal that males produce significantly higher levels of TNF-α (48) compared to their female counterparts (**Figure 4B**).

Additionally, although not statistically significant, there is a noticeable trend toward higher production of other pro-inflammatory cytokines, such as IFN-γ and IL-6 (48) in males (**Figures 4A, C**, respectively).

These elevated cytokine levels may play a crucial role in driving the inflammatory processes that characterize the MIS-C disease (1–5, 7–9, 11, 37).

These findings suggest that male patients may experience a stronger pro-inflammatory immune response, which could contribute to the observed sex-based differences in immune activation and disease severity in MIS-C (1–5, 7–9, 11, 37).

The analysis of correlations between T_R3–56_ cells and other immune cell populations in male and female MIS-C patients revealed that T_R3–56_ percentages negatively correlated with the percentage of CD45RA^+^ naïve T cells in males (**Table 2**).

**Table 2:** Correlation between T_R3–56_ or Treg cells and immune cells in male and female MIS-C patients.

% T_R3–56_ cells		
*Correlations in male*	Slope	*p* Value
% CD45 RA^+^ naïve T lymphocytes	-0,5385	0,0143
% Activated Th1 lymphocytes	0,5019	0,0286
*Correlations in female*	Slope	*p* Value
% CD45 RA^+^ naïve T lymphocytes	-0,7178	0,0107
% CD45 RO^+^ memory T lymphocytes	0,7289	0,0091

% Treg cells		
*Correlations in male*	Slope	*p* Value
% Activated Th1 lymphocytes	0,4956	0,0309
*Correlations in female*	Slope	*p* Value
% Th1 lymphocytes	-0,7801	0,0040
CD4/CD8 T cells ratio	0,6415	0,0276

Spearman’s rank correlation is reported as *Slope* and *p value*, as indicated.

Positive correlations with T_R3–56_ cells were observed for the percentage of activated Th1 cells in male MIS-C patients (**Table 2**).

In females, the percentage of T_R3–56_ cells negatively correlated with levels of CD45RA^+^ naïve T cells, while positively correlating with CD45RO^+^ memory T cells (**Table 2**).

These findings suggest a sex-specific role of T_R3–56_ cells in MIS-C. In males, T_R3–56_ negative correlation with CD45RA^+^ naïve T cells and positive association with activated Th1 cells indicate a role in immune Th1-driven responses. In females, the similar negative correlation between T_R3–56_ and CD45RA^+^ naïve T cells, along with their positive correlation with CD45RO^+^ memory T cells, may suggest a shift to immune memory.

These differences may reflect sex-related variations in immune adaptation and disease progression in MIS-C.

Treg cell percentages were positively associated with activated Th1 cells in male (**Table 2**).

Finally, Tregs negatively correlated with Th1 cells and positively with CD4/CD8 ratio in female MIS-C patients (**Table 2**).

These results suggest that the correlations between these two T regulatory populations and other immune cell populations differ between males and females, and may reflect sex-based differences in immune responses in MIS-C patients.

### 3.4 Stratification of MIS-C patients into two age groups (younger vs older children) and Sex differences within age groups in immune profile

Male and female MIS-C patients were categorized into two age groups: Group 1, comprising 26 children aged ≤9 years (18 males, 8 females), and Group 2, consisting of 13 children aged >9 years (7 males, 6 females). The selection of the cut-off of 9 years could constitute a limitation of the current study (see Study limitation paragraph).

In Group 1, 14 out of 26 children (53.8%) exhibited moderate to severe clinical conditions, with a higher prevalence in males (N=9) compared to females (N=5). Similarly, in Group 2, 8 out of 13 children (61.5%) experienced moderate to severe symptoms, with males (5 cases) again outnumbering females (N=3).

Although not statistically relevant due to the small sample size, an interesting trend emerges: among Group 1 patients with greater disease severity, males exhibited a higher mean percentage of T_R3–56_ cells (3.6 ± 2.2, mean ± SE) compared to females (0.8 ± 0.2). A similar pattern was observed in subjects with greater disease severity from Group 2, where males showed a higher mean percentage of T_R3–56_ cells (2.6 ± 0.7) than females (1.0 ± 0.3).

Furthermore, across both age groups, males appear to be at higher risk and tend to develop more severe clinical manifestations. This male predominance in MIS-C severity may be linked to biological factors such as hormonal differences, immune response variability, or genetic predispositions, which warrant further investigation.

Overall, our data suggest that age and sex are influential factors in MIS-C severity, with younger children and males being more susceptible to severe disease courses. These observations could have implications for risk stratification, clinical management, and therapeutic interventions in pediatric patients with MIS-C.

Statistically significant differences between groups were found in CD8^+^ T and CD45RA^+^ naïve T cells (**Supplementary Figure 3D** and **2L**, respectively), with both cell types being higher in Group 1 compared to Group 2. No differences in NK, B, T, CD4^+^ T lymphocytes, Th17 lymphocytes, Treg cells, T_R3–56_ cells, HLA-DR^+^ activated T, and CD45RO^+^ memory T cell percentages were observed between Group 1 and Group 2 (**Supplementary Figure 3**, other panels).

In Group 1, the elevated CD8+ T effector cell percentage may suggest a robust immune response in younger MIS-C patients, possibly involving T cell-mediated cytotoxicity to eliminate infected cells. While this adaptive response may serve to clear the pathogen and limit inflammation, it could also contribute to tissue damage characteristic of MIS-C. The increased presence of CD45RA^+^ naïve T cells may reflect their recruitment and activation in response to SARS-CoV-2 or associated inflammatory processes.

No differences in IFN-γ, TNF-α, IL-6, IL-10 and IL-17 production were observed between the two groups (data not shown).

To highlight sex-related differences in age-stratified groups, we assessed male and female MIS-C patients in Groups 1 and 2, aiming to identify variations across both younger and older children.

**Figure 5:**
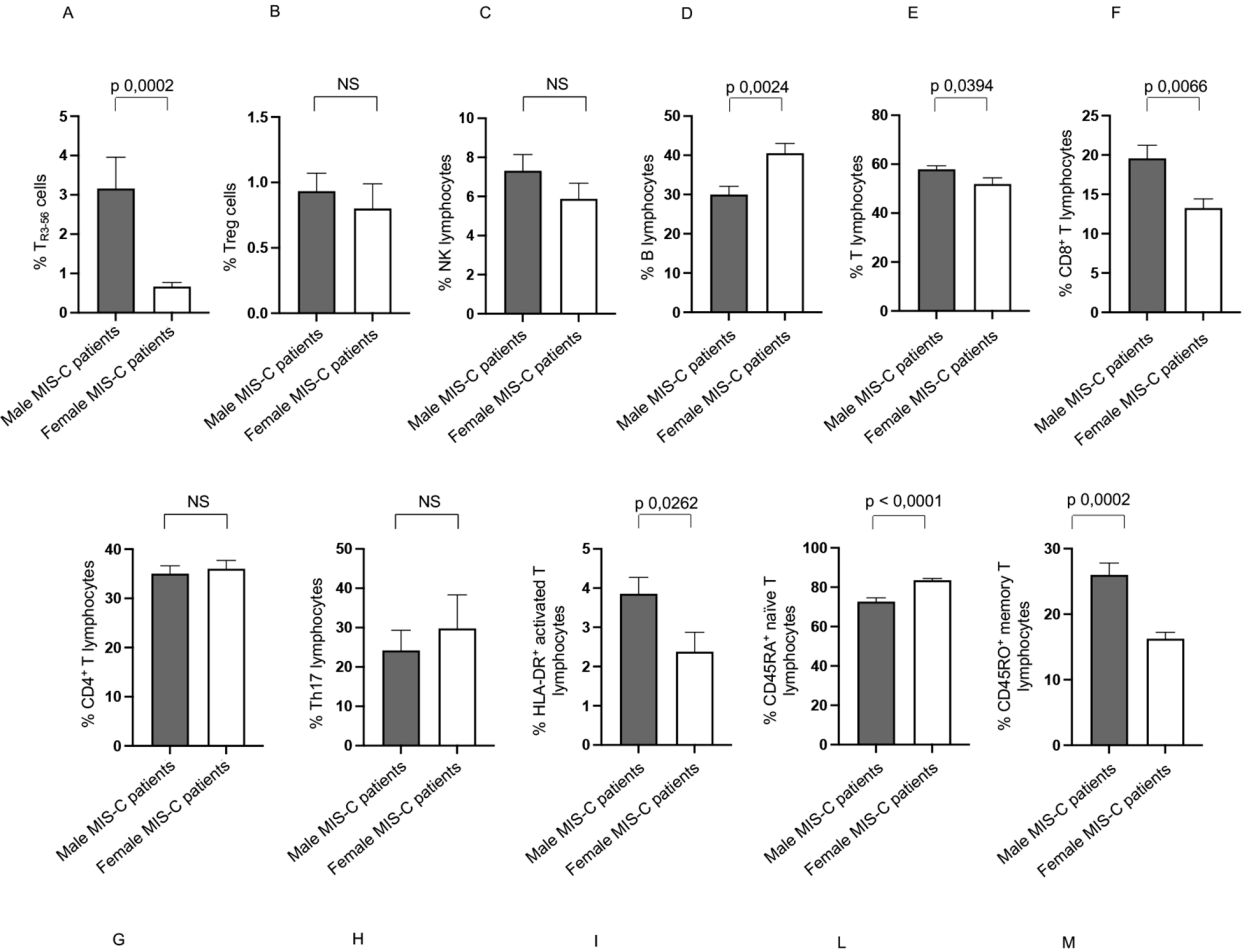
Differences between males and females in immune cells from Groups 1 patients. Analysis of T_R3-56_ **(A)**, Treg **(B)**, NK **(C)**, B **(D)**, T **(E)**, CD8+ T **(F)**, CD4^+^ T **(G)**, Th17 **(H)**, HLA-DR^+^ activated T **(I)**, CD45RA^+^ naïve T **(L)**, and CD45RO^+^ memory T **(M)** lymphocytes in male (gray bars) and female (white bars) of Group 1 patients. Cell percentages are reported in y axis *p* value is reported at the top of the bars. NS means not statistically significant.

In Group 1, males exhibited a higher percentage of T_R3-56_, T, CD8^+^ T, HLA-DR^+^ activated T and CD45RO^+^ memory T lymphocytes (**Figures 5A, E, F, I, M**, respectively), and a lower percentage of B and CD45RA^+^ naïve T lymphocytes (**Figures 5D, L**, respectively) compared to females.

Group 1 showed no significant sex-based variation in the percentages of Treg, NK, CD4^+^ T lymphocytes, and Th17 lymphocytes (**Figures 5B, C, G, H**, respectively).

**Figure 6:**
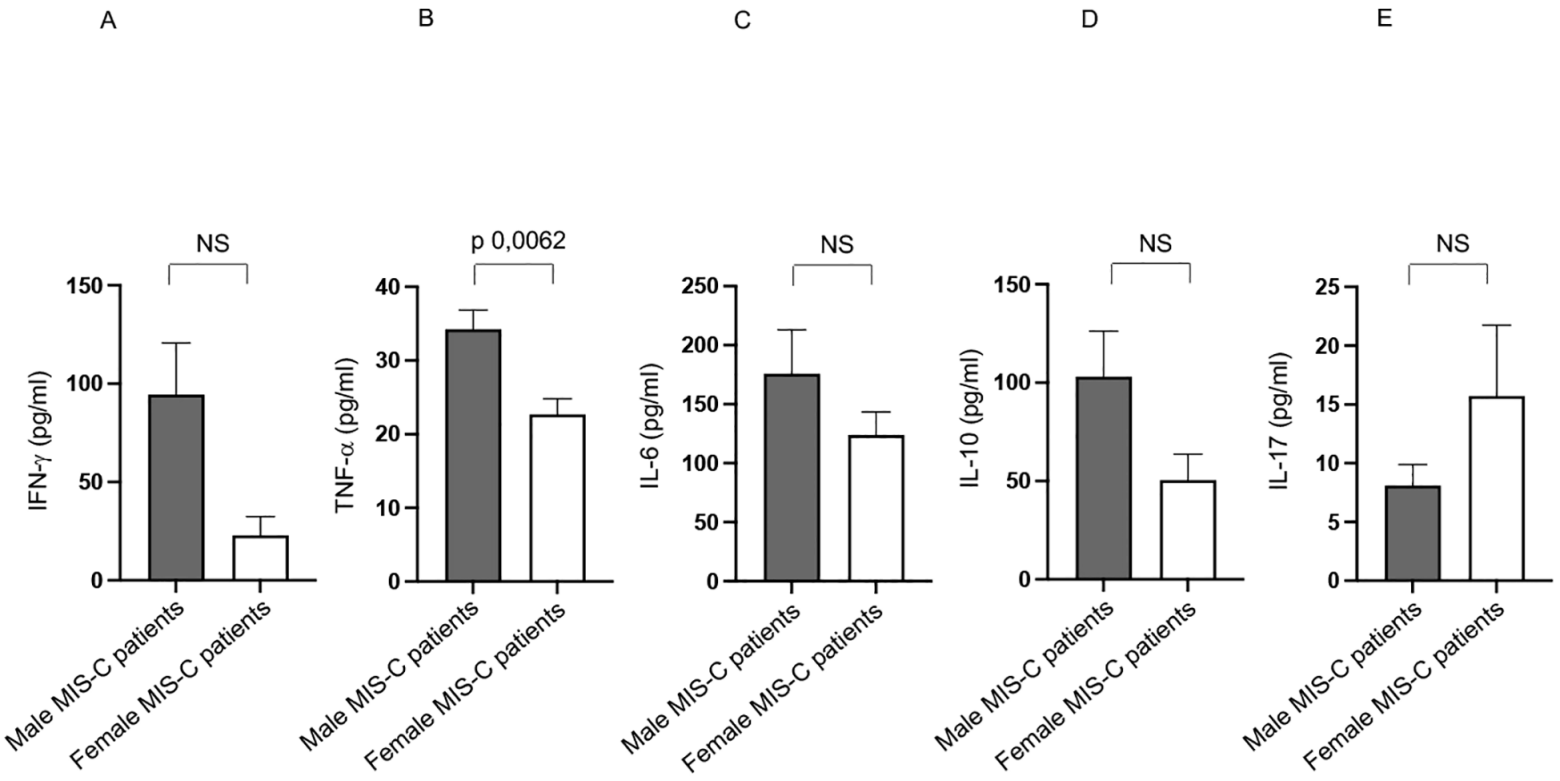
Differences between males and females from Group 1 patients in cytokine production. Analysis of INF-γ **(A)**, TNF-α **(B)**, IL-6 **(C)**, IL-10 **(D)** and IL-17 **(E)** production in male (gray bars) and female (white bars) from Group 1 patients. Cytokine amount values are reported in y axis. *p* value is reported at the top of the bars. NS means not statistically significant.

Intriguingly, males express a higher TNF-α production than females in Group 1 (**Figure 6B**), while other cytokines did not differ between males and females (**Figures 6A, C–E**).

Increased TNF-α level may contribute to increased systemic inflammation, tissue damage, and multisystem involvement. It could also be linked to more aggressive immune activation and the risk of a cytokine storm, a key feature of severe MIS-C cases (1–5, 7–9, 11, 37).

**Figure 7:**
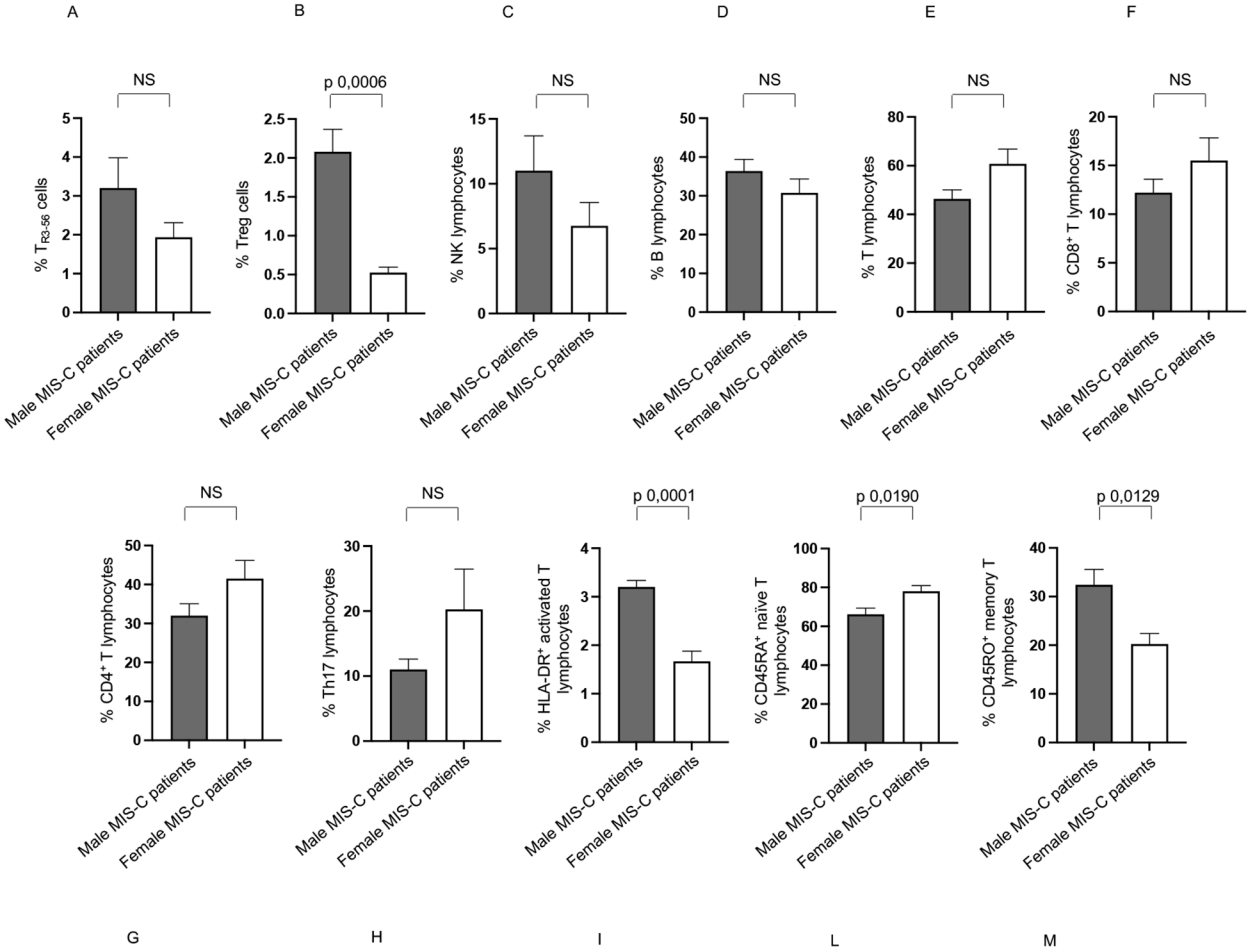
Differences between males and females in immune cells from Groups 2 patients. Analysis of T_R3-56_ **(A)**, Treg **(B)**, NK **(C)**, B **(D)**, T **(E)**, CD8^+^ T **(F)**, CD4^+^ T **(G)**, Th17 **(H)**, HLA-DR^+^ activated T **(I)**, CD45RA^+^ naïve T **(L)**, and CD45RO^+^ memory T **(M)** lymphocytes in male (gray bars) and female (white bars) of Group 2 patients. Cell percentages are reported in y axis*. p* value is reported at the top of the bars. NS means not statistically significant.

In Group 2, males exhibited a higher percentage of Treg, HLA-DR^+^ activated T, and CD45RO^+^ memory T lymphocytes (**Figures 7B, I, M**, respectively), and a lower percentage of CD45RA^+^ naïve T lymphocytes compared to females (**Figure 7L**).

No significant differences between males and females in Group 2 were found for T_R3-56_, NK, B, T, CD8^+^ T, CD4^+^ T, and Th17 cells (**Figures 7A, C–H**, respectively).

**Figure 8:**
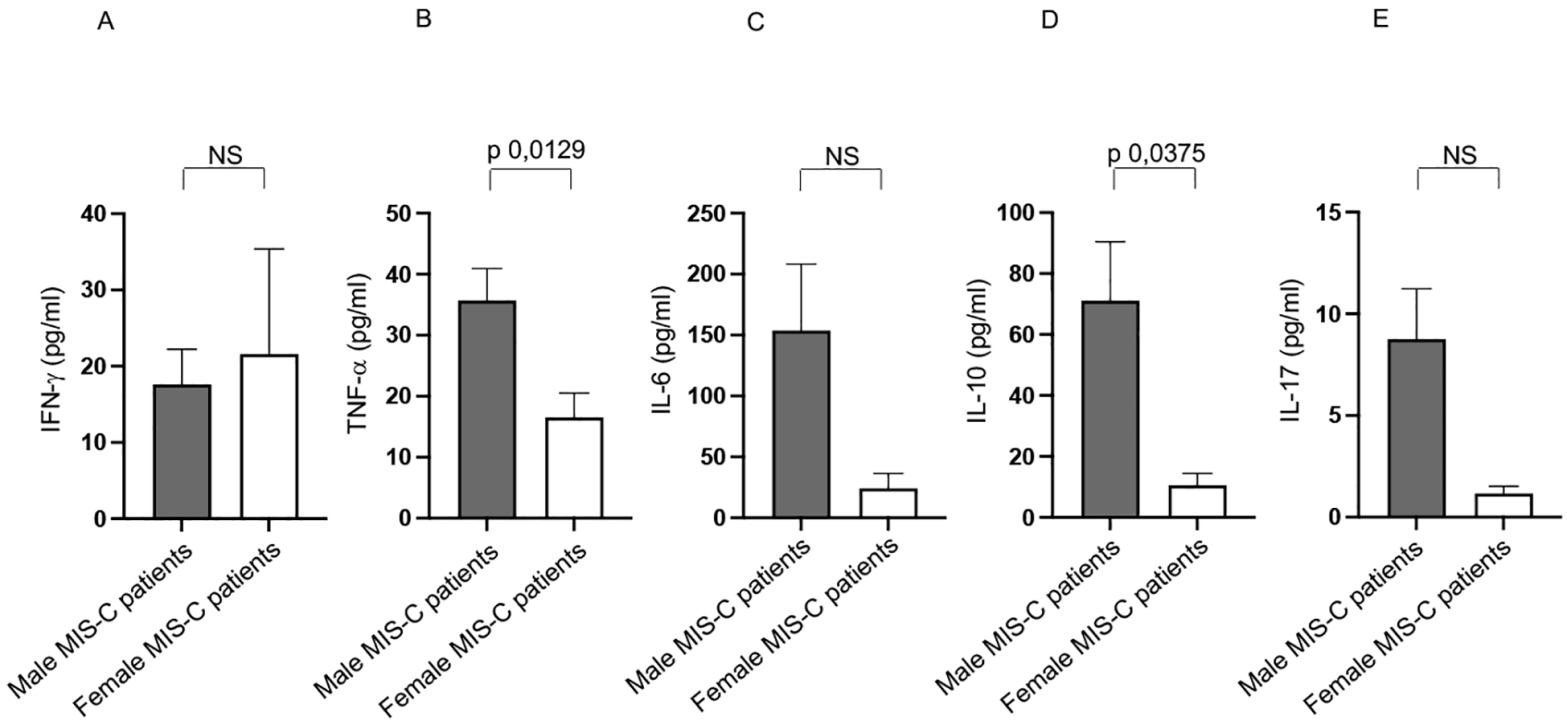
Differences between males and females from Group 2 patients in cytokine production. Analysis of INF-γ **(A)**, TNF-α **(B)**, IL-6 **(C)**, IL-10 **(D)** and IL-17 **(E)** production in male (gray bars) and female (white bars) from Group 2 patients. Cytokine amount values are reported in y axis. *p* value is reported at the top of the bars. NS means not statistically significant.

Similar to Group 1, male patients in Group 2 exhibited higher TNF-α production (**Figure 8B**). They also showed elevated IL-10 levels compared to female patients (**Figure 8D**).

No significant differences between sexes were observed for IFN-γ, IL-6, and IL-17 (**Figures 8A, C, E**, respectively).

### 3.5 Correlations between T_R3–56_ cells with cytokines and adaptive effector cells in males and females from groups 1 and 2

We assessed whether differences existed in the correlations between T_R3–56_ cells with cytokines and with the main effector cells of the adaptive immune response in male and female from Groups 1 and 2.

In this regard, the observation in males from Group 1 revealed that T_R3–56_ cells positively correlate with CD8^+^ T, CD45RO^+^ memory T, activated Th1 lymphocytes, and Treg cells (**Table 3**). Additionally, T_R3–56_ cells negatively correlate with CD4^+^ T cells, CD4/CD8 T cell ratio, B cells, CD45RA^+^ naive T lymphocytes in males from Group 1 (**Table 3**).

**Table 3:** Correlation between T_R3–56_ and cytokines or immune cells in males and females from Group 1 and Group 2 patients.

% T_R3–56_ cells								
	*Correlations in male*				*Correlations in female*			
	Group 1		Group 2		Group 1		Group 2	
	Slope	*p*-value	Slope	*p*-value	Slope	*p*-value	Slope	*p*-value
IFN-γ		NS	+0,8721	0,0024	+0,5181	0,0430		NS
TNF-α		NS	+0,9747	0,0001		NS	-1,000	0,0008
IL-6		NS		NS		NS	-0,8000	0,0381
IL-10		NS		NS		NS	-1,000	0,0008
IL-17		NS		NS		NS	-0,8000	0,0381
% CD4^+^ T	-0,4272	0,0147		NS		NS		NS
% CD8^+^ T	+0,3750	0,0344		NS		NS	+0,8000	0,0381
CD4/CD8 T ratio	-0,3658	0,0395		NS		NS	-1,000	0,0008
% B	-0,4491	0,0099	-0,7105	0,0290	-0,6606	0,0069		NS
% CD45RA^+^ naïve T	-0,4879	0,0062	-0,6669	0,0457		NS	-0,8000	0,0381
% CD45RO^+^ memory	+0,4287	0,0181		NS	+0,5273	0,0385	+0,8000	0,0381
% Th1		NS	+0,8208	0,0072	-0,5803	0,0210	+1,000	0,0008
% Th17		NS		NS	+0,6667	0,0062		NS
% Activated Th1	+0,4777	0,0102	+0,6882	0,0102		NS		NS
% Activated Th17		NS	-0,6669	0,0457		NS		NS
% Tregs	+0,4919	0,0058		NS		NS	-1,000	0,0008

Spearman’s rank correlation is reported as *Slope* and *p value*, as indicated.

These results suggest that in males from Group 1, T_R3–56_ cells are potentially associated with a more activated and sustained memory T cell response, as well as with cell regulatory immune profile.

T_R3–56_ cells positively correlate with IFN-γ, TNF-α, Th1 and activated Th1 lymphocytes (**Table 3**) in male patients of Group 2, suggesting an association with a pro-inflammatory and Th1-polarized immune response.

In females from Group 1, T_R3–56_ cells positively correlate with IFN-γ, CD45RO^+^ memory T and Th17 lymphocytes (**Table 3**). Conversely, T_R3–56_ cells negatively correlate with B and Th1 lymphocytes in females from the same of childhood patients in the same group (**Table 3**).

Therefore, T_R3–56_ cells appear to balance inflammation, shape memory, and modulate Th17 responses, supporting tolerance in Group 1 females, also suppressing B and Th1 cell activity.

In females of Group 2, T_R3–56_ cells positively correlate with cytotoxic CD8^+^ T cells, CD45RO^+^ memory T and Th1 lymphocytes (**Table 3**), suggesting a persistence of antigen-primed T cells.

In addition, T_R3–56_ cells negatively correlate with CD45RA^+^ naive T lymphocytes, Tregs, and the CD4/CD8 T cell ratio in Group 2 females (**Table 3**). Such inverse relationship may suggest a shift toward a more differentiated, antigen-experienced immune phenotype, with reduced immunosuppressive activity in females.

Finally, T_R3–56_ cells also negatively correlate with TNF-α, IL-6, IL-10 and IL-17 in female patients from Group 2 (**Table 3**). This suggests T_R3–56_ cells may contribute to a more controlled immune environment by limiting excessive inflammatory signaling.

## 4 Discussion

Our study aims to provide a deeper understanding of the immune mechanisms underlying MIS-C (1–5, 7–9, 11, 37), with a specific focus on immune cell dysregulation.

In this regard, we investigated the immunological characteristics of untreated MIS-C patients, with particular emphasis on the percentage of T_R3–56_ lymphocytes and their potential role in immune regulation within the context of MIS-C.

Our previous analysis identified significant immunological differences between MIS-C patients and healthy controls, including alterations in both pro-inflammatory and anti-inflammatory cytokines (IFN-γ, IL-6, IL-10, and TNF-α) and modifications in lymphocyte, monocyte, and granulocyte levels, along with changes in key innate and specific lymphocyte subpopulations highlighting a distinct immune dysregulation compared to healthy individuals (12).

We recently identified the T_R3–56_ cells as a novel regulatory T cell population in several immune-mediated diseases (28–32).

Here, we first highlighted that there are no statistically significant differences in the percentages of T_R3–56_ lymphocytes and Treg (19–27) cells between MIS-C patients and healthy controls. This evidence suggests that immune dysregulation in MIS-C may not be driven by changes in the abundance of these regulatory cells but rather by their functional roles in the interactions within the immune network.

In our observations, a positive correlation indicates that as one variable increases, the other also increases (or both decrease together), whereas a negative correlation implies that as one variable increases, the other decreases. Understanding these patterns is essential for interpreting the observed relationships between immune cell subsets and clinical parameters in MIS-C, as they may reflect coordinated or opposing biological processes.

In this regard, we observed a positive correlation between T_R3–56_ and Tregs in MIS-C patients, absent in controls. Such a noteworthy finding suggests a potential immunoregulatory function for T_R3–56_ in the disease context, which could indicate either a compensatory mechanism to counteract inflammation or a hallmark of immune dysregulation specific to MIS-C. This insight may potentially contribute to a better understanding of immune alterations in MIS-C and could have implications for future diagnostic and therapeutic strategies targeting immune regulation.

Moreover, our findings reveal distinct correlation patterns of T_R3–56_ cells with various immunological markers in MIS-C patients, providing novel insights into their potential role in immune modulation. These results highlight the importance of investigating functional dynamics beyond mere quantitative assessments to gain a deeper understanding of immune alterations in MIS-C.

In this regard, T_R3–56_ lymphocytes show negative correlations in MIS-C patients with CD45RA^+^ naïve T cells, which are crucial for immune responses and differentiation (44). Conversely, T_R3–56_ levels are positively correlated with CD45RO^+^ memory T cells (44).

These results suggest that T_R3–56_ cells may influence early immune activation and naïve T lymphocyte balance in MIS-C, linking them to antigen-primed T cells and long-term immunity (44).

T_R3–56_ also correlate with the activated Th1 cells (45) in MIS-C patients. This evidence suggests that T_R3–56_ cells may be involved in balancing immune responses, as their correlation with Tregs implies a potential role in immune tolerance and suppression of excessive activation, while their association with activated Th1 cells points to a contribution in pro-inflammatory responses.

This dual interaction may indicate a regulatory role in maintaining immune homeostasis, with T_R3–56_ cells potentially contributing to the fine-tuning of the balance between immune activation and suppression. Understanding this dynamic could provide insights into their role in immune-related diseases and inflammatory disorders.

This study aimed also to identify sex-based differences in MIS-C by analyzing immune activation and regulation in male and female patients.

Our findings confirm a male predominance in MIS-C, consistent with previous studies (1–5, 7–9, 11, 33–39). Moreover, disease severity was more frequently observed among males, who were more likely to present with moderate to severe clinical manifestations compared to females. However, our analysis indicates that these sex-related differences are not attributable to distinct severity patterns between males and females. Rather, the observed disparity is primarily explained by a higher overall incidence of MIS-C in males, resulting in a greater absolute number of male patients with severe presentations. Crucially, when disease severity was evaluated within each sex, no significant differences emerged in terms of clinical course or extent of organ involvement.

These findings suggest that male sex confers a greater susceptibility to developing MIS-C, but does not inherently predispose to a more severe disease phenotype. Collectively, our results support the notion of a sex-biased vulnerability to MIS-C, rather than a sex-specific variation in disease progression or severity.

Notably, male MIS-C patients exhibit significantly higher percentages of T_R3–56_ cells than females Interestingly, a positive correlation between T_R3–56_ and Tregs is observed in males but not in females. These findings suggest a potential sex-based difference in the immune response to MIS-C, with T_R3–56_ cells playing a more prominent role in males and may indicate a sex-specific immunoregulatory mechanism that warrants further investigation.

Male MIS-C patients show higher percentages of HLA-DR^+^ activated T (15–17, 45), and CD45RO^+^ memory T cells. The higher memory T cell levels in males correspond to a greater presence of CD45RA^+^ naïve T cells in females, highlighting potential sex-based differences in immune dynamics within MIS-C.

These findings suggest that males may have a more activated immune profile, whereas females might maintain a larger naïve T cell pool, potentially influencing disease progression and response to treatment.

We observed also a higher TNF-α production in male than in female MIS-C patients. Since such cytokine is a key mediator of inflammation (48), its increased levels in males could be associated with heightened immune activation, tissue damage, or a more severe clinical course.

These findings align with the observed greater activation of T cell subsets in males (15–17, 45), reinforcing the hypothesis that sex-related immune mechanisms influence MIS-C pathophysiology.

Sex-specific correlations suggest distinct immunoregulatory roles of T_R3–56_ cells. In males, their negative association with CD45RA^+^ naïve T cells and positive correlations with Tregs and activated Th1 cells point to involvement in immune differentiation and modulation of Th1-driven responses. In females, the negative correlation with naïve T cells and positive association with memory T cells imply a shift toward immune memory. Moreover, T_R3–56_ cells in males appear to contribute to balancing activation and regulation within a pro-inflammatory milieu, while in females, Treg correlations indicate stronger homeostatic control, potentially influencing immune adaptation and disease course in a sex-dependent manner. These data highlight sex-related variations in immune adaptation, which could influence disease progression and immune responses in MIS-C.

Moreover, we examined age-related differences in both male and female children to assess their potential impact on immune responses in MIS-C disease.

MIS-C patients were divided into two age groups: younger (Group 1) and older (Group 2) children, with males outnumbering females in both groups. In both age groups, a higher proportion of children exhibited moderate to severe clinical conditions, with males being more frequently affected than females. This trend was particularly evident in younger patients.

Group 1 patients exhibited a greater percentage of CD8+ T effector cells than Group 2. Such occurrence may point to a vigorous immune reaction, potentially utilizing T cell cytotoxicity to eradicate infected cells. This dual-edged adaptive response, while potentially beneficial for pathogen elimination and moderating inflammation, could also contribute to the tissue pathology described in MIS-C. The observed increment in CD45RA^+^ naïve T cells could be a sign of their engagement and activation triggered by SARS-CoV-2 infection or the consequent inflammatory environment in Group 1 patients.

Significant sex-based differences in lymphocyte populations were observed in Group 1 patients. Specifically, males exhibited a higher percentage of T, CD8^+^ T, T_R3-56_, HLA-DR_+_ activated T, and CD45RO^+^ memory T lymphocytes, and a lower percentage of B and CD45RA^+^ naïve T lymphocytes when compared to females. These results suggest that males show a T cell-dominant, activated/memory profile, while females have relatively higher B cell and naïve T cell percentages.

The consistently higher TNF-α production in males across both Group 1 and 2 indicates a potentially stronger inflammatory response in males than in females, contributing to the systemic inflammation characteristic of MIS-C.

The elevated percentages of HLA-DR^+^ activated T and CD45RO^+^ memory T cells, along with a decreased percentage of CD45RA^+^ naïve T cells in males of both MIS-C groups, suggest a more pronounced adaptive immune response, possibly reflecting prior antigen exposure or the development of robust immune memory.

The increase in T_R3–56_ cells in Group 1 and Treg cells in Group 2 suggests that males may be differentially regulating their immune responses compared to females, likely maintaining a distinct balance between immune activation and control.

In addition, males with greater disease severity consistently appeared to present a trend with higher T_R3–56_ cell percentages than females. This observation suggests a potential sex-related difference in immune response, warranting further investigation into the role of T_R3–56_ cells in MIS-C pathophysiology.

The higher percentages of CD8^+^ T cells and HLA-DR^+^ activated T cells in males, particularly in younger MIS-C patients (Group 1), suggest a more robust cytotoxic immune response, possibly driven by a heightened immune activation or a response to SARS-CoV-2 infection. The increased percentage of CD45RO^+^ memory T cells in younger males indicates that male MIS-C patients may exhibit a more pronounced adaptive immune response than in females, potentially due to earlier antigen exposure or stronger immune memory development. A decrease in B cells suggests a shift toward a more T-cell-mediated immune response in younger males compared to females in Group 1.

Finally, we assessed whether differences existed in the correlations between T_R3–56_ cells with cytokines and with the main effector cells of the adaptive immune response in male and female from Groups 1 and 2.

In males from Group 1, T_R3–56_ cells show a positive correlation with CD8^+^ T cells, memory T cells, activated Th1 lymphocytes, and Tregs, indicating a potential link to an activated and sustained memory T cell response, along with a regulatory immune profile. Conversely, T_R3–56_ cells negatively correlate with CD45RA^+^ naive T cells, B cells, and the CD4/CD8 T cell ratio, suggesting a shift away from these immune cell effectors.

In the Group 2, T_R3–56_ cells correlate positively with inflammatory markers such as IFN-γ, TNF-α, Th1, and activated Th1 lymphocytes, indicating an association with a pro-inflammatory, Th1-polarized immune response (45, 48).

In females from Group 1, T_R3–56_ cells positively correlate with IFN-γ, memory T cells, and Th17 lymphocytes (45, 48), while negatively correlating with B cells and Th1 lymphocytes. This evidence suggests that T_R3–56_ cells play a role in balancing inflammation, shaping memory, and modulating Th17 responses to support immune tolerance, while suppressing B and Th1 cell activity.

In females from Group 2, T_R3–56_ cells correlate positively with cytotoxic CD8+ T cells, memory T cells, and Th1 lymphocytes, suggesting the persistence of antigen-primed T cells.

However, T_R3–56_ cells negatively correlate with naive T cells, Tregs, and the CD4/CD8 T cell ratio, which may indicate a more differentiated, antigen-experienced immune phenotype with reduced immunosuppressive activity. Additionally, negative correlations with inflammatory cytokines such as TNF-α, IL-6, IL-10, and IL-17 (48) suggest that T_R3–56_ cells may contribute to a more controlled immune environment by limiting excessive inflammatory signaling.

These findings highlight the complex role of T_R3–56_ cells in shaping immune responses, with distinct patterns observed between sexes and age groups.

In males, T_R3–56_ cells appear to be actively involved in both immune activation and regulation. Specifically, these cells interact with Tregs and activated Th1 cells, which may contribute to a heightened inflammatory response as well as immune regulation. This dual role could be critical in modulating the immune system during MIS-C, potentially influencing the severity and progression of the disease.

In contrast, in females, T_R3–56_ cells seem to primarily support immune memory and regulatory responses, with an emphasis on controlling inflammation. These cells may play a more suppressive role, helping to maintain immune tolerance by modulating Th17 responses and limiting excessive activation of inflammatory pathways. This function could be crucial in preventing the excessive immune response that characterizes MIS-C, particularly in the more regulated immune environment observed in females.

## 5 Conclusion

Taken in all, our findings underscore the complexity of the immune response in MIS-C, emphasizing the relevance of understanding the differential roles of T_R3–56_ cells. The distinct functions of these cells in males and females highlight a potential sex-specific immunological framework that may contribute to differences in disease susceptibility, severity, and progression. Given the observed sex-based disparities in immune profiles, further research into the mechanisms underlying these variations appears to be essential. This could lead to the development of targeted, sex-specific therapeutic strategies that more effectively address the unique immune responses in each group.

Moreover, these results point to the potential for T_R3–56_ cells to serve as biomarkers or therapeutic targets in MIS-C, offering new avenues for intervention. Further studies exploring the precise signaling pathways and molecular interactions involving T_R3–56_ cells could provide deeper insights into their role in immune dysregulation and disease manifestation. Ultimately, understanding how these cells influence immune responses in MIS-C could pave the way for more tailored, effective treatments that account for both sex-based differences and the unique immune mechanisms involved in this complex inflammatory syndrome.

## 6 Study limitations

The study did not assess the functional aspects of the analyzed cells, so the hypotheses drawn from immunophenotypic profiles lack proof of concept. Additionally, due to practical constraints, long-term follow-up of pediatric MIS-C patients was not feasible, limiting the ability to track changes or outcomes over an extended period. These factors should be considered when interpreting the findings of this study.

Moreover, a limitation of our study is the lack of SARS-CoV-2 variant characterization, which restricts the generalizability of our findings and highlights the need for future research accounting for variant-specific effects on MIS-C.

Another limitation of the present study is the arbitrary selection of age 9 as the cut-off for patient stratification. Although this choice is grounded in developmental and immunological hypotheses -recognizing that puberty-related hormonal changes, particularly in females, typically begin around this age and may influence immune function and disease susceptibility - it remains a pragmatic threshold rather than a definitive biological boundary. This arbitrary selection may impact the interpretation of puberty-related sex differences in MIS-C and should be considered when generalizing the findings.

Finally, a limitation of this study is the relatively small size of the control group, which included only 13 pediatric healthy subjects. However, the enrollment of a larger number of healthy children was constrained by the critical emergency situation during the COVID-19 pandemic, which limited access and availability for study participation.

## Data Availability

The raw data supporting the conclusions of this article will be made available by the authors, without undue reservation.

## Supplementary material

Supplementary Figure 1 Representative flow cytometry plots illustrating the gating strategy employed. Lymphocytes were initially gated as region 1 (R1) based on side scatter area (SSC-A) versus forward scatter area (FSC-A) **(A)**. Subsequent analyses of cell subsets were performed within the R1 gate. T_R3–56_ cells were identified as double-positive for CD3 and CD56 (**B**; upper right quadrant). NK cells were defined as CD3^-^CD56^+^ (**B**; lower right quadrant). CD3^+^ T cells were identified as CD3^+^CD56^-^ (**B**; upper left quadrant). CD8^+^ T cells were gated as CD3^+^CD8^+^ (**C**; upper right quadrant). B cells were defined by CD19^+^CD3^-^ expression (**C**; right quadrant). The identification of additional cell populations, as described in the main manuscript, was performed using comparable gating strategies. The percentage of cells within each population is indicated in the respective dot plot quadrants. FSC-A: forward scatter area; SSC-A: side scatter area; FSC-H: forward scatter height.

Supplementary Figure 2 Analysis of Natural Killer **(A)**, B **(B)**, T **(C)**, CD8+ T **(D)**, CD4+ T **(E)**, and Th17 **(F)** lymphocytes in male and female MIS-C patients. Cell percentages are reported in y axis*. p* value is reported at the top of the bars. NS means not statistically significant.

Supplementary Figure 3 Analysis of NK **(A)**, B **(B)**, T **(C)**, CD8+ T **(D)**, CD4+ T **(E)**, and Th17 **(F)**, Treg **(G)**, T_R3-56_ **(H)**, HLA-DR^+^ activated T **(I)**, CD45RA+ naïve T **(L)**, and CD45RO+ memory T **(M)** lymphocytes in Group 1 (horizontally striped bars) and Group 2 (white bars). Cell percentages are reported in y axis *p* value is reported at the top of the bars. NS means not statistically significant.
