# Supplementary figures and images for: Sex-based immunological differences in multisystem inflammatory syndrome in children: potential role of TR3–56 cells for pathogenesis, diagnosis, and therapy
Source: Front Immunol. 2025 Jun 20;16:1606115. doi: 10.3389/fimmu.2025.1606115 (PMC12226297; doi:10.3389/fimmu.2025.1606115)

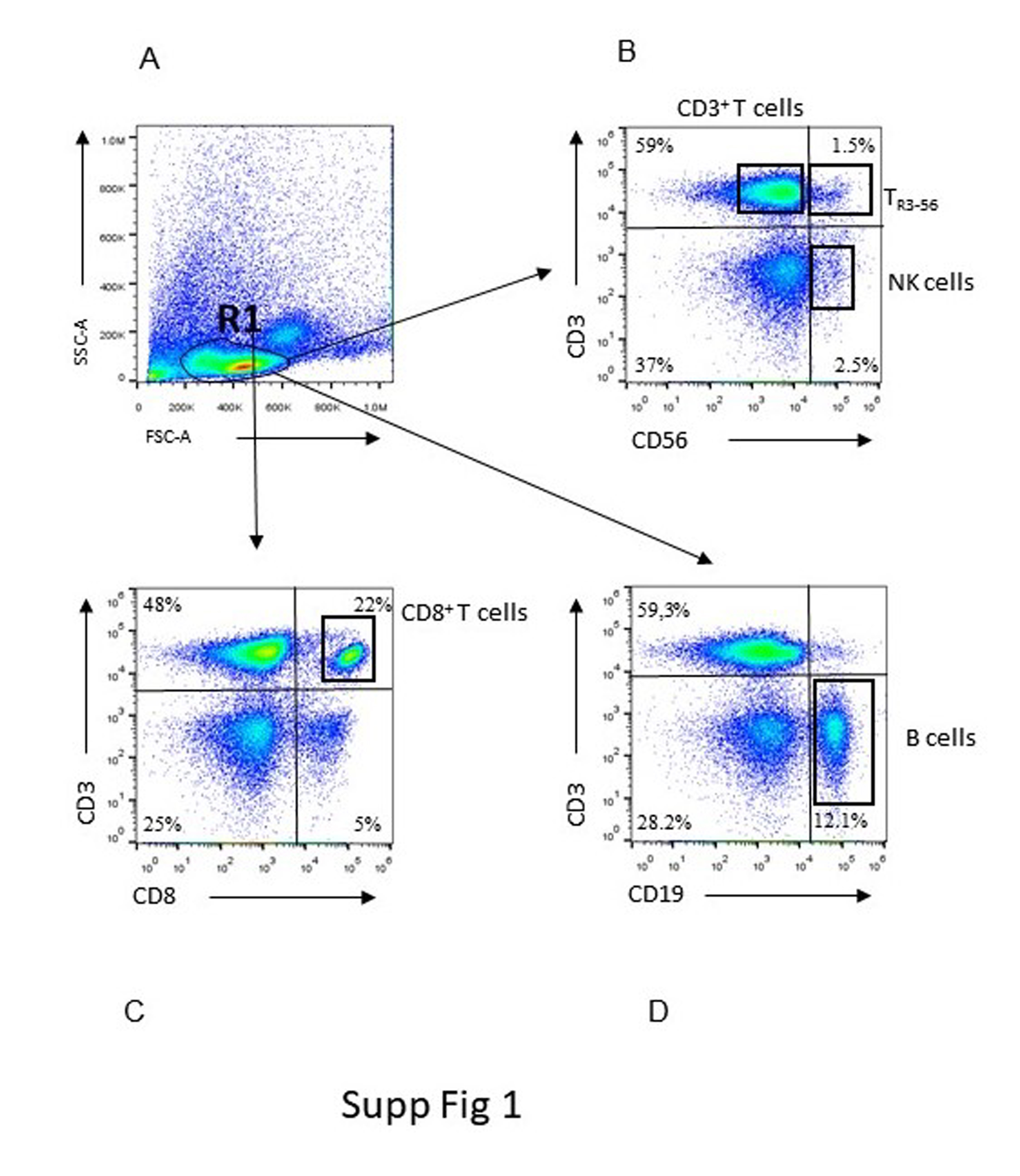

Supplement: Supplementary Figure 1 — Representative flow cytometry plots illustrating the gating strategy employed. Lymphocytes were initially gated as region 1 (R1) based on side scatter area (SSC-A) versus forward scatter area (FSC-A) (A). Subsequent analyses of cell subsets were performed within the R1 gate. TR3–56 cells were identified as double-positive for CD3 and CD56 (B; upper right quadrant). NK cells were defined as CD3-CD56+ (B; lower right quadrant). CD3+ T cells were identified as CD3+CD56- (B; upper left quadrant). CD8+ T cells were gated as CD3+CD8+ (C; upper right quadrant). B cells were defined by CD19+CD3- expression (C; right quadrant). The identification of additional cell populations, as described in the main manuscript, was performed using comparable gating strategies. The percentage of cells within each population is indicated in the respective dot plot quadrants. FSC-A: forward scatter area; SSC-A: side scatter area; FSC-H: forward scatter height. [file Image1.jpeg]

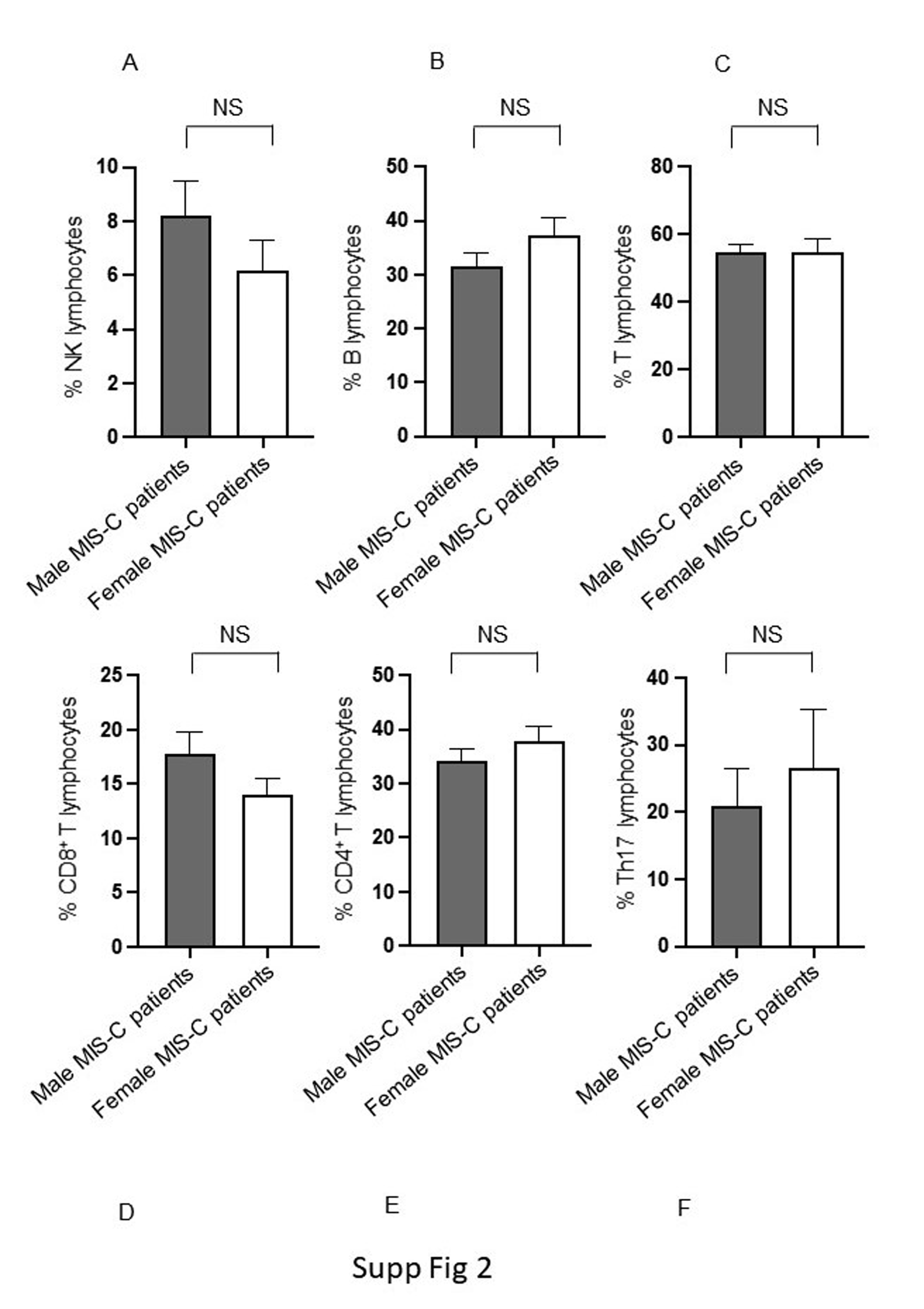

Supplement: Supplementary Figure 2 — Analysis of Natural Killer (A), B (B), T (C), CD8+ T (D), CD4+ T (E), and Th17 (F) lymphocytes in male and female MIS-C patients. Cell percentages are reported in y axis. p value is reported at the top of the bars. NS means not statistically significant. [file Image2.jpeg]

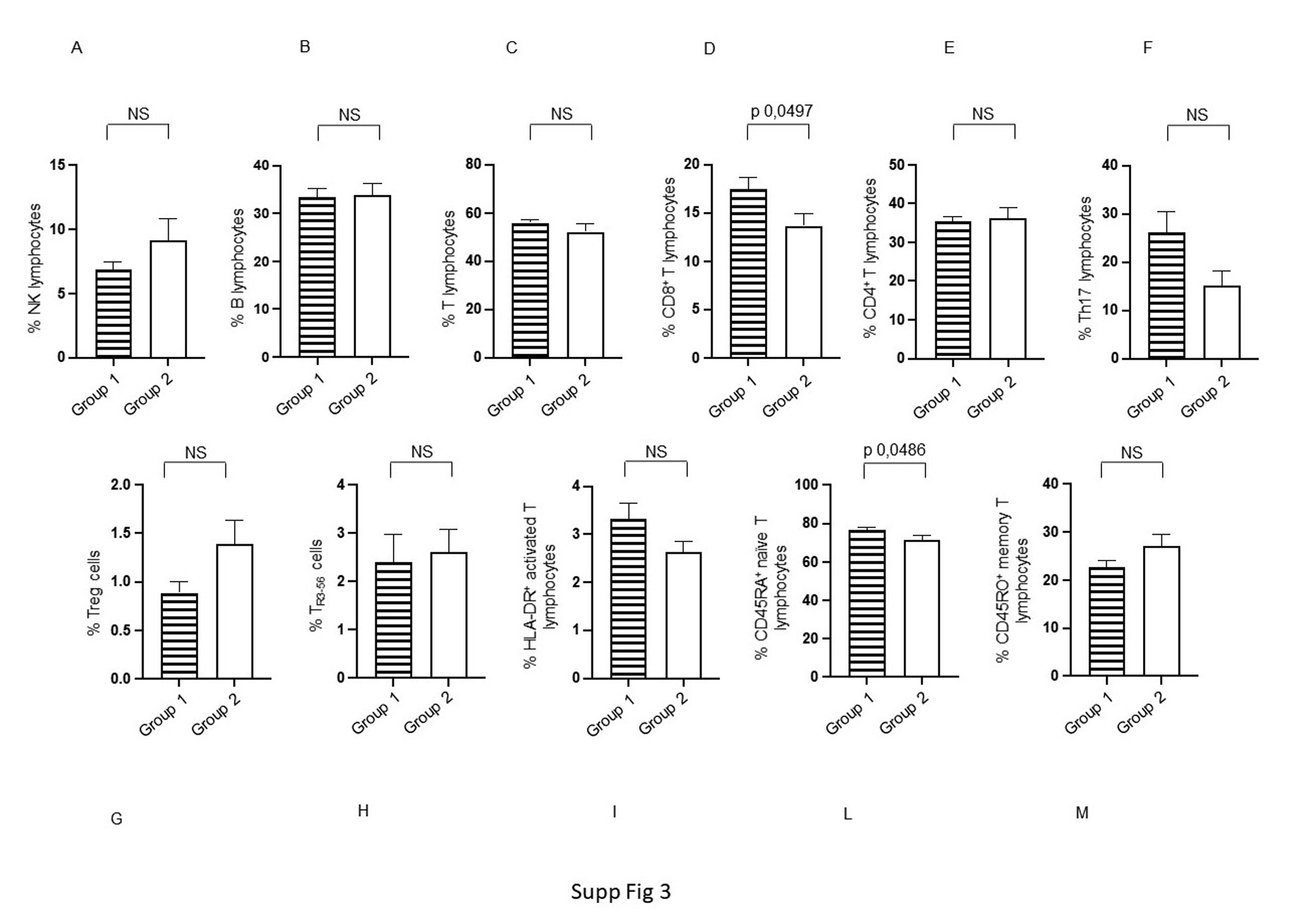

Supplement: Supplementary Figure 3 — Analysis of NK (A), B (B), T (C), CD8+ T (D), CD4+ T (E), and Th17 (F), Treg (G), TR3-56 (H), HLA-DR+ activated T (I), CD45RA+ naïve T (L), and CD45RO+ memory T (M) lymphocytes in Group 1 (horizontally striped bars) and Group 2 (white bars). Cell percentages are reported in y axis p value is reported at the top of the bars. NS means not statistically significant. [file Image3.jpeg]
