# Supplementary material for: Sex-based immunological differences in multisystem inflammatory syndrome in children: potential role of TR3–56 cells for pathogenesis, diagnosis, and therapy
Source: Front Immunol. 2025 Jun 20;16:1606115. doi: 10.3389/fimmu.2025.1606115 (PMC12226297; doi:10.3389/fimmu.2025.1606115)
Supplement: Supplementary file 4 [file Table1.docx]

**Supplementary Table 1.**

|  | | |
| --- | --- | --- |
| **Surface markers of lymphocyte populations** | | |
| **Lymphocyte population** | **Surface markers** |  |
| T | CD3^+^ |  |
| CD4^+^ T | CD3^+^ CD4^+^ |  |
| CD8^+^ T | CD3^+^ CD8^+^ |  |
| HLA-DR^+^ activated T | CD3^+^ HLA-DR^+^ |  |
| Treg | CD3^+^ CD127^-^ CD4^+^ CD25^++^ |  |
| T_R3-56_ | CD3^+^ CD56^+^ |  |
| Th17 | CD3^+^ CD4^+^ CD196^+^ CD183^-^ |  |
| Th1 | CD3^+^ CD4^+^ CD196^-^ CD183^+^ |  |
| Naïve T | CD3^+^ CD45RA^+^ |  |
| Memory T | CD3^+^ CD45RO^+^ |  |
| B | CD19^+^ |  |
| NK | CD56^+^ |  |
| **Monoclonal Antibodies (MoAb) and Fluorochromes** | | |
| **MoAb** | **Fluorochrome** | **Producer** |
| CD3 | PACIFIC BLUE | Beckman Coulter |
| CD4 | PeCy5.5 | Beckman Coulter |
| CD8 | APC-Alexafluor750 | Beckman Coulter |
| CD19 | APC-Alexafluor750 | Beckman Coulter |
| CD25 | PC-7 | Invitrogen |
| CD56 | PC-7 | Beckman Coulter |
| CD45RA | FITC | Invitrogen |
| CD45RO | FITC | Invitrogen |
| CD127 | FITC | Becton Dickinson |
| CD183 | PE | Becton Dickinson |
| CD196 | PC-7 | Becton Dickinson |
| HLA-DR | HV-500 | Becton Dickinson |
|  | | |
